# Amide Proton Solvent Protection in Amylin Fibrils Probed by Quenched Hydrogen Exchange NMR

**DOI:** 10.1371/journal.pone.0056467

**Published:** 2013-02-15

**Authors:** Andrei T. Alexandrescu

**Affiliations:** Department of Molecular and Cell Biology, University of Connecticut, Storrs, Connecticut, United States of America; University of Leeds, United Kingdom

## Abstract

Amylin is an endocrine hormone that accumulates in amyloid plaques in patients with advanced type 2 diabetes. The amyloid plaques have been implicated in the destruction of pancreatic β-cells, which synthesize amylin and insulin. To better characterize the secondary structure of amylin in amyloid fibrils we assigned the NMR spectrum of the unfolded state in 95% DMSO and used a quenched hydrogen-deuterium exchange technique to look at amide proton solvent protection in the fibrils. In this technique, partially exchanged fibrils are dissolved in 95% DMSO and information about amide proton occupancy in the fibrils is determined from DMSO-denatured monomers. Hydrogen exchange lifetimes at pH 7.6 and 37°C vary between ∼5 h for the unstructured N-terminus to 600 h for amide protons in the two β-strands that form inter-molecular hydrogen bonds between amylin monomers along the length of the fibril. Based on the protection data we conclude that residues A8-H18 and I26-Y37 comprise the two β-strands in amylin fibrils. There is variation in protection within the β-strands, particularly for strand β1 where only residues F15-H18 are strongly protected. Differences in protection appear to be due to restrictions on backbone dynamics imposed by the packing of two-layers of C2-symmetry-related β-hairpins in the protofilament structure, with strand β1 positioned on the surface and β2 in the interior.

## Introduction

Type 2 diabetes affects over 300 million people worldwide, with the incidence of the disease expected to reach over 500 million by 2030 [1]. Insulin resistance and high blood glucose levels characterize the disease but its causes are multi-factorial [2], [3]. One of the hallmarks of advanced type 2 diabetes is the development of amyloid plaques consisting of the endocrine hormone amylin (also known as islet amyloid polypeptide or IAPP) [4]. The amyloid plaques have been implicated in the destruction of pancreatic β-cells that synthesize both amylin and insulin [3], [4]. As with other amyloid diseases it is unclear whether fibrils or soluble oligomers are responsible for amylin pathology [5]–[8]. Even if fibrils are not the main culprits, their properties are important to understand since they could serve as a reservoir from which toxic oligomers dissociate [9].

The structure of amylin fibrils has been characterized by solid-state nuclear magnetic resonance (ssNMR) [10], electron paramagnetic resonance (EPR) [11], two-dimensional infrared spectroscopy (2DIR) [12] and cryo-electron microscopy (cryo-EM) [10], [11], [13]. The consensus from these studies is that the amylin monomers adopt a hairpin structure composed of two β-strands in the fibrils. Each of the β-strands forms an intermolecular parallel β-sheet pairing with the equivalent β-strand from an adjacent amylin monomer. Two stacks of β-hairpins related by C_2_-symmetry run in opposite directions along the length of the fibril and pack against each other to form the protofilament building block of the fibrils [10]. As with other amyloid fibrils, more subtle aspects of the structure are less clear and show larger differences between models obtained by different techniques. These include the precise sequence limits of the β-strands, the domain-swap stagger of the β-strands, the twist of the β-strands with respect to the fibril axis, and the organization of the foundational cross-β-sheet into higher-order structure [10]–[12], [14].

Hydrogen exchange (HX) protection provides information on the location and stability of protein secondary structure. When a protein is dissolved in deuterium oxide (D_2_O), amide protons exchange with deuterons at rates determined by intrinsic factors such as pH, temperature, and the protein sequence [15]. HX can be slowed markedly when amide protons are involved in hydrogen-bonded structure that makes them inaccessible to solvent [16]. Consequently, HX data can identify amide protons involved in secondary structure and probe structural stability [17]. While solution nuclear magnetic resonance (NMR) studies of proteins are usually limited to proteins and complexes with molecular weights below 30–50 kDa, quenched hydrogen exchange (qHX) experiments can circumvent this size limit by transferring information on amide proton occupancy to the denatured state [18], [19]. In the qHX experiment, HX is initiated by suspending amyloid fibrils in D_2_O. After varying periods of time, HX is quenched by flash freezing. The partially exchanged fibril samples are then lyophilized and dissolved in a strongly denaturing solvent such as 95% dimethyl sulfoxide (DMSO). The DMSO solvent serves two purposes. First, DMSO is sufficiently chaotropic to unfold most types of amyloid fibrils to monomers. Second, because DMSO is an aprotic solvent, HX from the denatured state occurs on timescales of hours compared to minutes or seconds in H_2_O, allowing the detection of amide protons trapped in the fibril.

The qHX technique was first described for model amyloid fibrils formed by the *Escherichia coli* protein CspA. Since the method was first published [18] it has been used to study a number of amyloid fibrils relevant to human disease [9], [20]–[26]. These include β-microglobulin [21], Aβ [22], [24], α-synuclein [25], prion protein [20], cystatin [23] and apolipoprotein [26]. Here, qHX is used to investigate amyloid fibrils formed by amylin. The pattern of amide proton protection in amylin fibrils is consistent with the location of the two β-strands in structural models from ssNMR [10], except the protection data suggests the strands are slightly longer, with strand β2 extending further into the ‘amyloidogenic segment’ consisting of residues S20 through S29 [27], [28]. Protection is less consistent with an alternative model derived from EPR data [11]. Strand β1 shows less extensive protection than β2, an observation that appears to be related to the supramolecular packing of β-sheets, with strand β2 buried in the center of the protofilament structure and β1 exposed on the surface. Molecular dynamics (MD) simulations based on the ssNMR model of amylin fibrils, are used to test the hypothesis that increased motional flexibility accounts for the decreased amide proton protection observed for strand β1.

## Materials and Methods

### Materials

Recombinant ^15^N-amylin was purchased as a lyophilized powder from rPeptide (Bogart, GA). The peptide was expressed in *Escherichia coli* and has an intact C2–C7 disulfide bond but differs from human amylin by not having an amidated C-terminus, which is an enzymatic post-translational modification in mature human amylin [4]. D_2_O (isotope purity >99.96%) and DMSO-d_6_ (99.96%) were from CIL (Andover, MA). Dichloroacetic acid (DCA) was from Aldrich (St. Louis, MO) and deuterated dichloroacetic acid: Cl_2_CDCO_2_D, 99.7% (d_2_-DCA) was from CDN Isotopes (Point-Claire, Quebec, Canada).

### Control Experiments to Demonstrate the Solubility of Amylin Fibrils in DMSO

Three control experiments were done to verify that amylin fibrils are soluble in DMSO and to optimize the conditions for the qHX experiments. (**1**) To start, 0.1 mg lyophilized, un-fibrillized ^15^N amylin was dissolved in 220 µl 95% DMSO/5% DCA at an apparent pH measured in DMSO (pH*) of 3.5, to give an amylin concentration of 0.12 mM. The heteronuclear single-quantum correlation (^1^H-^15^N HSQC) spectrum obtained at 25°C showed that amylin is soluble, monomeric, unfolded, and thus amenable to NMR spectroscopy. The spectrum showed no changes after 1 month at room temperature, demonstrating amylin is stable in 95% DMSO. Additional pulse-field gradient translational diffusion NMR experiments [29] showed that amylin in DMSO has an apparent hydrodynamic radius of 15±1 Å, close to the expected value of 17 Å for an unfolded monomer (Figure S1). (**2**) Next, it was determined that negligible amounts of ^15^N-amylin monomers remain in solution when amylin undergoes fibrillization, and that lyophillization does not disrupt the fibrils. A 0.12 mM ^15^N-amylin sample in H_2_O buffer containing 10 mM sodium phosphate pH 7.4 with 10% (v/v) acetonitrile was fibrillized without agitation for 4 days at 37°C. Electron microscopy (EM) images of fibrils grown under these conditions are shown in Figure S2. Amylin fibrils were sedimented at 15,000 g for 30 min. The supernatant, and pellet resuspended in H_2_O, were flash-frozen in a dry ice/ethanol bath and lyophilized. No NMR signals from amylin were observed when the lyophilized supernatant or the lyophilized fibrils were resuspended in H_2_O. This indicated that negligible amounts of monomeric amylin remained in the supernatant, and that species with molecular weights detectable by NMR did not dissociate from the fibrils during lyophilization. (**3**) In marked contrast, NMR signals were detected when the experiment was repeated, and the lyophilized pellet was taken up in 95% DMSO/5% DCA rather than water. The 95% DMSO solvent is able to dissolve fibrils to unfolded amylin monomers, giving a two-dimensional (2D) ^1^H-^15^N HSQC spectrum and ^15^N-edited 1D spectrum (Figure S3) comparable to that obtained when un-fibrillized amylin is dissolved in 95% DMSO. It has been previously reported that amylin fibrils are insoluble in DMSO [28], [30]. Unlike the naturally occurring hormone the ^15^N-labeled amylin used in this work is not amidated at its C-terminus, which may increase the solubility of fibrils in DMSO. A second important difference is that the fibrils used in this work were prepared from a pure preparation of amylin, whereas in the previous study [30] amylin fibrils were isolated from a pancreatic tumor where they may have been associated with cofactors [31] that could affect stability and solubility in DMSO.

### Amylin Fibrillization and Quenched Hydrogen Exchange Experiments

A 1.4 mg sample of ^15^N-amyin was dissolved in 140 µl of acetonitrile to disrupt any preexisting aggregates, and taken up in 1.26 ml of 20 mM sodium phosphate buffer, pH 7.4. The resulting amylin concentration for fibrillization was 250 µM. The final concentration of acetonitrile in the fibrillization buffer was 10% (v/v). A concentration of 0.02% NaN_3_ (w/v) was added to prevent bacterial growth during fibrillization. Following dissolution, the solution was sonicated continuously for 1 minute at 75% power to break up any potential aggregates. To form fibrils, the sample was incubated at 37°C without agitation in a low-retention Eppendorf tube for 116 h (∼5 days). Fibrils were collected by sedimentation for 45 min at 15,000 g in an Eppendorf desktop micro-centrifuge.

The pellet of approximately 40 µl volume was resuspended in 1.24 ml of 99.96% D_2_O and the pH of the suspension was determined to be 7.6. The H_2_O/D_2_O dilution factor for was ∼31-fold, corresponding to a final concentration of at most 3% H_2_O in the sample. For the hydrogen-deuterium exchange reaction, the sample was maintained at 37°C in an EchoTherm IN30 incubator from Torrey Pines Scientific (Carlsbad, CA).

To monitor HX, 0.2 ml aliquots were withdrawn at seven time points: 0.08, 1, 8, 24, 73, 99 and 356 h. The fibril suspension in D_2_O was mixed for 30 s with a Fisher Vortex Genie-2 before each aliquot was withdrawn. The aliquots were immediately frozen in a dry ice/ethanol bath, lyophilized, and stored at −80°C until use. For NMR experiments, the partially exchanged lyophilized fibrils were dissolved in 0.5 ml of 95% d_6_-DMSO/5% d_2_-DCA. Note that deuterated d_2_-DCA was used for NMR experiments to prevent back-exchange of protons from the acid to amylin. The pH of each sample was checked after the NMR experiments and was pH* 3.4±0.1.

### NMR Spectroscopy

Unless otherwise noted, a 600 MHz Varian Inova instrument equipped with a cryogenic probe was used for all NMR experiments. NMR assignments for ^15^N-amylin in 95% DMSO/5% DCA at a temperature of 25°C and pH* 3.5 were obtained from 3D TOCSY-HSQC (70 ms mix time) and 3D NOESY-HSQC (250 ms mix time) experiments. Assignments have been deposited in the BioMagResBank (BMRB) under accession number 18795.

Amide proton HX in the fibrils was read out from the lyophilized partially exchanged aliquots dissolved in 95% d_6_-DMSO/5% d_2_-DCA using 2D ^1^H-^15^N HSQC spectra recorded at a temperature of 25°C. The d_6_-DMSO signal was used for the deuterium lock. The 2D ^1^H-^15^N HSQC spectra were collected with 1024 complex points in the ^1^H dimension and 32 complex points in the ^15^N dimension. Spectra were typically acquired with 16 transients averaged per free induction decay for a total acquisition time of 21 minutes. The NMR data were processed and ^1^H-^15^N crosspeak heights were measured using the iNMR software package (Mestrelab Research).

### Gaussian Network Model Calculations using the ssNMR Model of Amylin Fibrils

Two models of the amylin fibril structure satisfy the ssNMR data: 4eql24930x2 and 4eql5432x2 [10]. The models differ with respect to the β-strand two-residue periodicity that determines which residues face the interior and exterior of the amylin β-hairpin fold [10]. Except where noted, the 4eql5432x2 model was analyzed, since this model is supported by EPR spin-label mobility data on amylin fibrils [11]. Theoretical B-factors based on the Gaussian Network Model (GNM) algorithm were calculated from the amylin fibril coordinate files with the oGNM online server [32], using a Cα-Cα cutoff distance of 10 Å.

## Results and Discussion

### Amylin Fibrils Show Variable Amide Proton Exchange Protection

**Figure 1:**
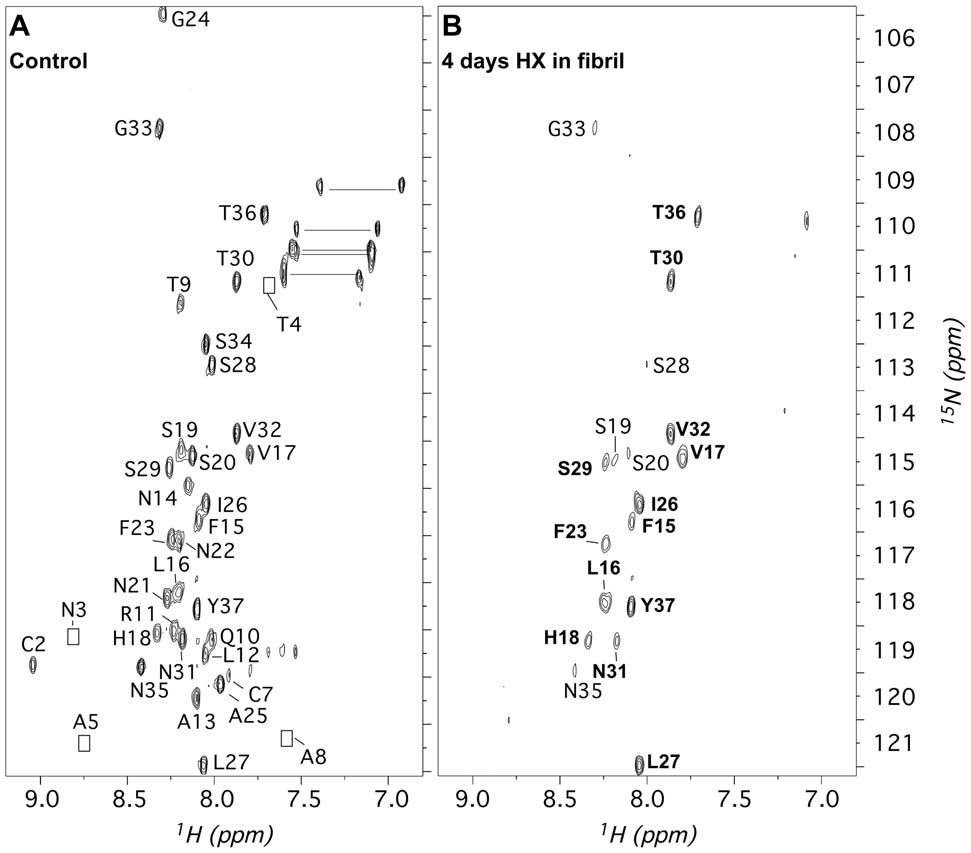
^1^H-^15^N HSQC spectra illustrating hydrogen exchange in amylin fibrils. (**A**) Control spectrum of unfibrillized ^15^N-amylin freshly dissolved in 95% d_6_-DMSO/5% DCA at 25°C, pH 3.5. Backbone crosspeaks are labeled according to sequence-specific assignments. Residues N3, T4, A5, and A8 are only visible at lower contours than shown. The group of crosspeaks connected by horizontal lines between 109 and 111 ppm (^15^N) are unassigned sidechain amide groups from the 6 Asn and 1 Gln in amylin. (**B**)Spectrum of a ^15^N-amylin after 4 days (99h) of D_2_O exchange in the fibril state, recorded in 95% d_6_-DMSO/5% d_2_-DCA. Strongly protected amide protons are labeled in bold type.

Figure 1 compares spectra of fully protonated amylin (Fig. 1A) with amylin partially exchanged in fibrils grown from an aqueous solution containing 10% (v/v) acetonitrile (Fig. 1B). NMR assignments for amylin in 95% DMSO/5% DCA were obtained for all 36 of the expected ^1^H-^15^N backbone amide correlations, except residue T6. The first eight residues show weaker ^1^H-^15^N crosspeaks than the rest of the peptide (Fig. 1A). Weaker correlations from this region were also seen for ^15^N-amylin in H_2_O [31] and SDS micelles [33], suggesting NMR line-broadening associated with an intrinsic dynamic process such as conformational exchange involving the C2–C7 disulfide bond.

Figure 1B shows the spectrum of ^15^N-amylin in DMSO after 4 days of D_2_O exchange in the fibrils. The spectrum is plotted at contour levels that emphasize residues with the strongest amide proton protection, which are labeled in bold type. Most of the strongly protected amide protons are within the two β-strands identified in the ssNMR model. The protected residues that lie immediately outside of the β-strands, H18 and I26–L27, suggest that the β-strand limits extend beyond those identified for the ssNMR model. Residues labeled in plain type show intermediate amide proton occupancy. Most of these residues also fall within the two β-strands, pointing to variability in protection within a given element of secondary structure. The residues with the weakest protection are either not seen, or close to the baseline noise in the spectrum after 4 days of D_2_O exchange. These include residues in the N21-A25 turn between the β-strands and residues C2–C7, which are disordered in the ssNMR model of amylin. Interestingly, the segment A8–A13 that forms the N-terminal portion of strand β1 in the ssNMR model is also weakly protected. Note that in the fibril the β-strands form two intermolecular β-sheets [10], with possibly independent stabilities.

**Figure 2:**
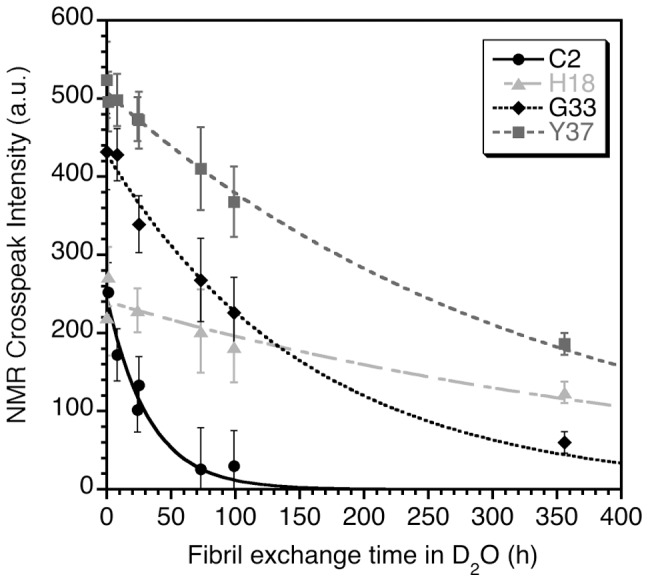
Representative solvent exchange kinetics for amide protons in amylin fibrils. Error bars were estimated from the average root-mean-square baseline noise of the ^1^H-^15^N HSQC spectra. The curves are fits of amide proton intensity decay data to an exponential model: y = I_0_•exp(-τ•x), obtained using the program KaleidaGraph v 4.1.3 (Synergy Software). The two free variables in the fits were I_0_, the initial amplitude and τ, the time constant for exchange.

Hydrogen exchange in amylin fibrils was characterized at seven time points ranging from 5 min to 356 h (∼14 days). Figure 2 shows amide proton intensity decay data for four representative residues. The amide proton of residue C2, which is in the unstructured N-terminus of amylin, exchanges with a fast rate. Residue G33, in strand β2 of the amylin fibril model exchanges with an intermediate rate. Amide protons that exchange with slow rates are represented by H18 and Y37, the C-terminal residues in strands β1 and β2. The observed differences in exchange rates between residues within the same strand (e.g. G33 and Y37 from strand β2), suggests that structural stability varies within a given element of secondary structure, as is often found in folded globular proteins [17], [34].

### Interpretation of Protection in Terms of the Amylin Fibril Structure

**Figure 3:**
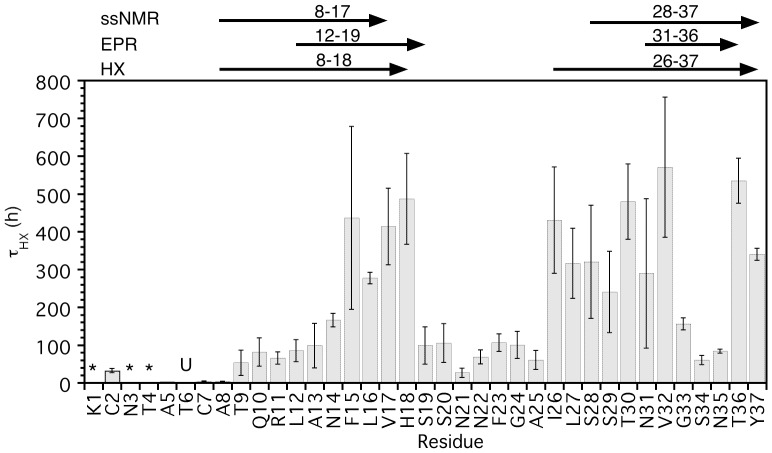
Time constants for hydrogen exchange as a function of residue position in the sequence. The top of the figure indicates the position of the two β-strands reported for the ssNMR [10] and EPR models of the amylin fibril structure, as well as the revised secondary structure limits based on the qHX data in this work. Uncertainties in exchange time constants were estimated from standard errors of the fits of the qHX data to exponential decays (Fig. 2). The symbols ‘*’ indicate amide protons that exchange with rates too fast to measure, ‘U’ indicates that the amide proton of T6 is unassigned.

Figure 3 shows time constants for exchange, determined for each residue from least-squares fits of amide proton decay data to an exponential model (Fig. 2). The largest time constants between 300 and 600 h are found for amide protons within, or immediately adjacent to the two β-strands (Fig 3). At the next level of protection, time constants between 50 and 150 h occur in the turn between the two β-strands but also for residues T9-N14 in the N-terminal part of strand β1 and for residues G33-N35 in strand β2. The fastest exchange is seen for residues K1-C7 at the N-terminus of the peptide, which are disordered in the amylin fibril structure [10]–[12]. The β-strand limits reported for the ssNMR [10] and EPR [11] models of amylin fibrils, together with those inferred from the HX results in this work are indicated at the top of Fig. 3.

**Figure 4:**
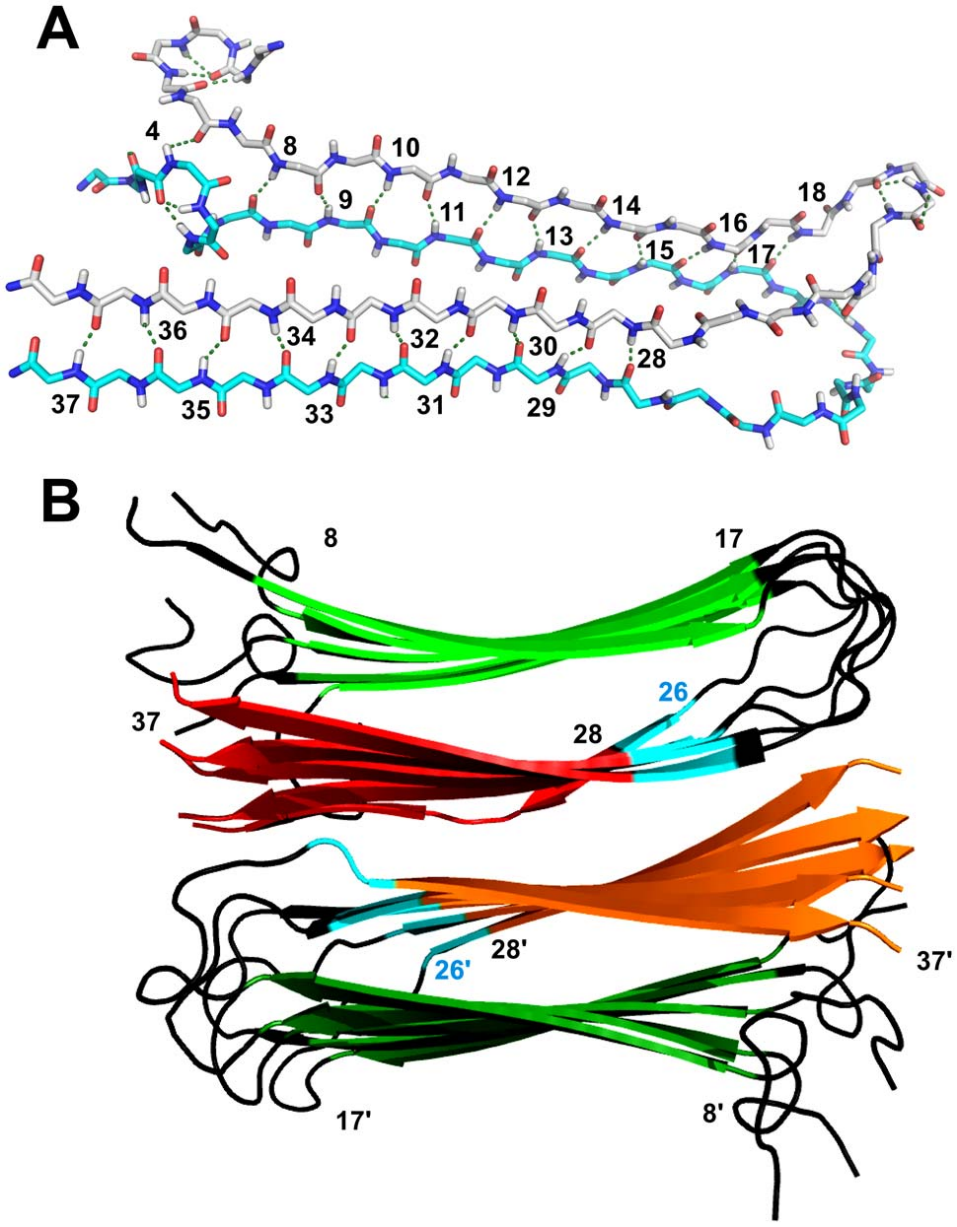
The ssNMR structural model of amylin fibrils [**10**]. The long axis of the fibrils runs in and out of the plane of the page. (**A**) Backbone hydrogen bonding between two adjacent amylin monomers in the fibril. Amide protons involved in intermolecular β-sheet hydrogen bonds are labeled alternatively in the blue and gray monomers. Note that the β-sheet hydrogen bonding is continuous along the length of the fibril, so that the amide proton of T36 in the blue monomer is a hydrogen bond donor for the carbonyl of S35 in the next monomer below (not shown). (**B**) In the ssNMR model of amylin fibrils two columns of amylin β-hairpins stack against each other with C_2_ symmetry to form a protofilament [10]. The C-terminal strands (red and orange) constitute the packing interface between the two layers of β-sheets, whereas the N-terminal strands (green) are on the surface. Residues I26-L27 which were not assigned to strand β2 in the ssNMR model but which nevertheless show strong qHX protection are colored in light blue. The drawings were rendered in PyMOL [39].

The ssNMR model [10] of the amylin protofilament (Fig. 4) consists of ten amylin monomers, packed into two columns of five monomers that are related by C_2_ rotational symmetry. Figure 4A illustrates the intermolecular β-sheet hydrogen bonding between two adjacent monomers stacked along the fibril axis. Figure 4B shows the packing of the two columns of β-hairpins. The C-terminal strands β2 are on the inside of the protofilament, while the N-terminal strands β1 are on the outside. The protection data obtained for amylin fibrils (Fig. 3) is in overall agreement with the ssNMR model (Fig. 4) but there are some important exceptions. First, H18 is protected even though it is just outside the 8–17 limits reported to form strand β1 [10]. Residue H18 was restrained to form β-sheet hydrogen bonds in the ssNMR structure calculations [10], its secondary chemical shift predicts that it is in a β-sheet conformation [10], and its amide protons serve as a hydrogen-bond donors to V17 from adjacent monomers in 62% of the amylin monomers that constitute the amylin fibril ssNMR model. In the ssNMR model, H18 falls in the β-sheet region of Ramachandran space in 9 of the 10 monomers that make up the fibril. These observations suggest that H18 should be included as the last residue in strand β1. H18 is an important residue, since its ionization state is critical in determining the pH dependence of fibrillization [35] and because replacement of H18 with positively charged arginine reduces amylin toxicity [36].

For the second β-strand, the qHX results suggest that hydrogen-bonded structure starts at I26, two residues earlier than the N-terminus reported for strand β2 in the ssNMR model, S28 [10]. The primary data used to restrain residues in β-sheet conformations in the ssNMR structure calculations [10] were predictions from the TALOS program which assigns secondary structure based on secondary chemical shift differences from random coil values [37]. The TALOS program [37], and the newer version TALOS+ [38], have become the standards for deriving backbone torsional angle restraints for NMR structure calculations of soluble proteins. Nevertheless, the original TALOS program had an error rate of incorrect secondary structure assignment of 3% [38]. The TALOS prediction based on the ssNMR chemical shifts of amylin fibrils suggest that L27 is not in a β-sheet conformation but otherwise support β-sheet structure for all residues between G24-T36 (c.f. Supplementary Table 1 of [10]). Except for residue L27, the ssNMR chemical shift data could be consistent with the N-terminus of strand β2 starting at G24 and the C-terminus of strand β1 ending at residue S20. While strong protection is not seen for any of the residues in the S19-G24 segment, this need not preclude β-sheet structure as residues A8-N14 in strand β1 and G33-N35 in strand β2 are weakly protected (Fig. 3). In terms of the structural models based on the ssNMR data, residues I26-L27 have dihedral angles that fall well within the β-sheet region of Ramachandran plots in 10 out of 10 structures. This is also evident for the PyMol [39] generated ribbon diagram of the ssNMR amylin fibril model in Fig. 4B, where residues I26-L27 are indicated in light blue and are identified by the program as belonging to a β-sheet structure based on their dihedral angles. Dihedral angles that fall outside of the β-sheet region are not seen until residues N21-G24 in the ssNMR models. The distinguishing feature of the I26-L27 segment in the ssNMR model is that it does not form β-sheet hydrogen bonds unlike the rest of the residues S28-Y37 in strand β2. In NMR structures, residues are typically restrained to form hydrogen bonds based on HX protection data. While it is possible that the HX protection observed herein for I26-L27 is due to burial of these residues in the core of the structure rather than β-sheet hydrogen bonding, that ssNMR chemical shifts are also consistent with β-sheet structure suggests that this segment is part of strand β2. Inclusion of the I26-L27 segment as the beginning of strand β2 would lead to better packing interactions against the C-terminal end of strand β1 and packing against the C-terminal end of strand β2 from C2-symmetry-related monomers than irregular structure (Fig. 4B). The extension of strand β2 further into the ‘amyloidogenic segment’ [27] to I26, could also better explain the behavior of the I26P mutation of amylin, which greatly reduces fibril formation and inhibits fibril formation by the WT sequence in *trans* [40]. The structural analysis described above was done for the 4eql54324x2 ssNMR model but also holds true for the alternative 4eql24930x2 model.

An alternative model of amylin fibrils has recently been calculated based on EPR data [11]. The largest difference between the EPR and ssNMR models is the ‘domain-swapped’ out-of-plane stagger of the two β-strands, which spans three peptide layers in the EPR model [11] compared to the hairpin fold of amylin monomers in the ssNMR model [10]. There are also differences in the limits of the β-strands between the ssNMR and EPR models. The limits of secondary structure in the EPR investigation were identified based on two types of data: (1) a two-residue periodicity in the mobility of introduced spin-labels that is characteristic of the inside-outside polarity of sidechains in a β-strand, and (2) a characteristic distance of ∼21 Å between spin-labels introduced with an i to i+6 sequence spacing in a β-strand. In the EPR model strand β1 is comprised of residues L12-S19 and β2 of N31-T36. The later start of strand β1 is a result of the increased mobility of the A8-R11 segment in the EPR data [11]. Increased mobility for this segment is also observed by ssNMR [10]. The end of strand β1 at S19 in the EPR model is consistent with the strong protection observed for H18 and the inclusion of this residue in strand β1 in the present study. Strand β2 in the EPR model (N31-T36) ends one residue earlier and starts three residues later than in the ssNMR model (S28-Y37), whereas the HX protection data in this work suggests that strand β2 begins as early as I26. In contrast to strand β1, there was only one probe of i to i+6 distances reported for strand β2, between residues G24 and T30. The distance between these probes was 23 Å, indicating a conformation more extended than the expected 21 Å distance [11], which seems consistent with a β-sheet conformation. The only mobility probe available between residues 25 and T30 was for residue S28, so that these data also do not rule out an earlier starting position for strand β2. The inclusion of residue Y37 as the last residue in strand β2 is supported by strong HX protection, and fluorescence data indicating restricted mobility and solvent accessibility for Y37 as well as FRET contacts to residues F15 and F23 [41].

### Comparison with Flexibility Predictions from Molecular Dynamics Calculations

The beginning of strand β1 comprised of residues A8–A13 shows minimal HX protection, with slowly exchanging amide protons only observed for residues N14-H18 (Fig. 3). The lack of protection for the N-terminal part of strand β1 indicates this segment is flexible. These results are consistent with ssNMR line broadening noted for residues A8–A13 in 2D ^13^C fpRFDR (finite-pulse radiofrequency-driven recoupling) spectra of amylin fibrils [10]. Line broadening in NMR spectra is typically associated with motion on µs-ms timescales. Fast motion on these µs-ms timescales would provide an avenue for amide proton exchange on the much slower hour to day timescales of the HX experiments in this work. Increased mobility of the A8–A13 segment also agrees with EPR data for amylin fibrils. Residues A8–A13 show increased EPR line-widths characteristic of increased mobility, and reduced differences in the mobility of spin-labels introduced on the inside and outside of the β-sheet in the segment spanning positions A8–A13 (Fig. 2 in [11]).

**Figure 5:**
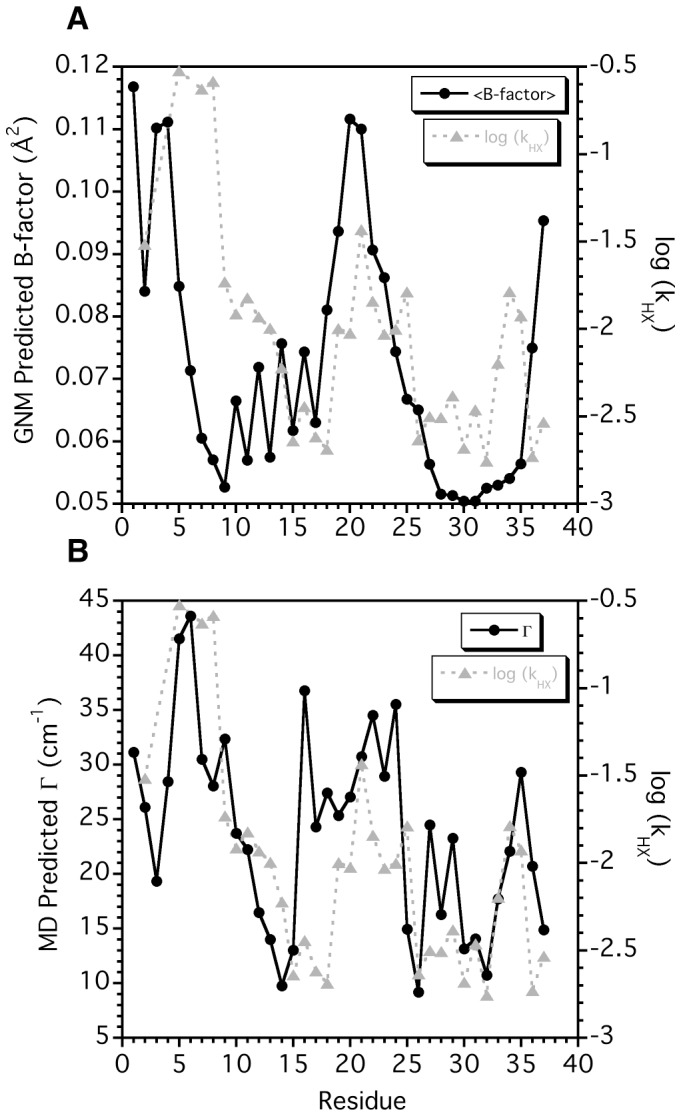
Comparison of experimental HX rates obtained in this work (gray symbols) with theoretical simulations of amylin fibril flexibility (black symbols). (**A**) Theoretical B-factors obtained from a GNM calculation [32], [42] of protein dynamics based on the ssNMR model of amylin fibrils [10]. The B-factors were averaged over the 10 amylin monomers in the ssNMR model [10]. (**B**) Predicted 2DIR lineshapes (Γ_i_) for amylin fibrils calculated from a MD simulation of the ssNMR amylin fibril structural model. The Γ_i_ data are from Fig. 9 of reference [12].

To test the hypothesis that the lower qHX protection observed for strand β1 is due to its position on the surface of the protofilament (Fig. 4B), GNM calculations [32], [42] of protein flexibility were performed using the ssNMR model of the amylin protofilament [10]. The GNM formalism models fluctuations about a mean structure as dependent on the distribution of distance contacts to nearby Cα atoms [42]. The predicted amplitudes of fluctuations at different sites can be used to calculate theoretical B-factors [42], which for native proteins have been shown to be in good agreement with experimental B-factors determined by X-ray crystallography and to correlate with HX protection factors [34], [42]–[44]. The theoretical B-factors calculated for the amylin fibril model are shown by the black symbols in Fig. 5a. The GNM calculations predict small B-factors indicative of reduced mobility for strands β1 and β2, as well as larger B-factors for the N-terminal strand β1 compared to the C-terminal strand β2. Although the GNM calculations capture the features of the HX sequence profile (gray symbols in Fig. 5A) the quantitative correlation to the observed HX rates is poor (R-value = 0.17, ρ = 0.3 for n = 33).

A better agreement (Fig. 5B) is seen when the HX rates are compared to theoretically predicted inhomogeneous frequency contributions to the 2DIR diagonal linewidths of amylin fibrils, Γ_i_ [45], calculated from an all-atom MD simulation [12] of the solvated ssNMR amylin fibril model. The Γ_i_ values were obtained by taking into account the fluctuating electric fields at a given site caused by the movement of all nearby atoms in the MD simulation. The Γ_i_ and log(k_HX_) data in Fig. 5B are pair-wise correlated with an R-value of 0.56 (ρ<0.001 for n = 33). The Γ_i_ values show a gradient of decreasing flexibility from the unstructured segment ending at C7 to about residue N14 in strand β1, in good agreement with the qHX data. The biggest differences occur for residues L16-H18 where the MD calculations over-predict flexibility compared to the HX data. The turn segment between the two β-strands has large HX rates and Γ_i_ values. A spike is seen for both the theoretical Γ_i_ values and the experimental HX rates near residues G33-N35 in strand β2, before both values fall at the C-terminus of amylin. Although the origin of the disorder for residues G33-N35 is unknown, experimental support for increased flexibility has been observed by 2DIR spectroscopy [12].

### Conclusions

The two β-strands that form the hydrogen-bonding network between monomers in ssNMR [10] and EPR [11] models of the amylin fibril structure show the greatest HX protection. Overall the agreement between the sequence-position limits of the β-strands in the ssNMR model and the HX data is good, except that the HX data suggests that strand β1 extends by one residue to H18 and strand β2 starts two residues earlier at L26. Differences in protection are observed within each β-strand, much like in native proteins. In the case of amylin fibrils these differences correlate with the packing of β-sheets into the higher-order protofilament structure. The N-terminal strand β1 on the surface of the protofilament, shows weak protection until the last five residues. By contrast, amide protons are protected throughout the C-terminal strand β2, which is buried in the protofilament structure. The HX studies described herein set the foundation for investigations to determine if protection in fibrils accrues through intermediates or arises in an all-or-none fashion, to look at how fibril structure changes with solution variables such as pH or when complexed with accessory molecules (e.g. metals or glycosaminoglycans) and to determine binding sites for ligands and drugs that target fibril growth.

## Supporting Information

Figure S1 **NMR experiments demonstrate that amylin is an unfolded monomer in DMSO.** (**A**) 1D-^1^H NMR spectrum of 220 µM human amylin (with an amidated C-terminus) in 95% d_6_-DMSO/5% d_2_-DCA, pH* 3.5, 25°C. The large resonances at 2.5 and 6.7 ppm are due to residual natural abundance DMSO and DCA, respectively. The methyl resonance at 0.8 ppm was used to characterize amylin diffusion. (**B**) Pulse-field gradient measurements of amylin translational diffusion. Experiments were carried out on a Bruker 500 MHz spectrometer with 1,4-dioxane added as an internal standard to the sample in A. From the diffusion coefficients of dioxane and the peptide we can calculate a hydrodynamic radius of 15±1 Å for amylin, using the formula R_peptide_ = (D_dioxane_/D_peptide_)R_dioxane_ and assuming a hydrodynamic radius of 2.12 Å for dioxane. The expected hydrodynamic radius for an unfolded protein is given by the empirical equation R_h_ = (2.21±1.07)N^0.57±0.02^, where N is the number of residues. The predicted (17 Å) and experimental (15±1 Å) values are close, indicating that amylin behaves as an unfolded monomer in DMSO.
(TIF)

Figure S2 **Electron micrograph of amylin fibrils.** Fibrils of recombinant ^15^N-amylin were formed under the same conditions as the hydrogen exchange experiments. Fibrils were transferred to a 400-mesh carbon-coated grid, rinsed with H_2_O, and negatively stained with 1% uranyl acetate. Images were obtained on a FEI Tecnai G^2^ BioTWIN instrument that is part of the UConn electron microscopy facility.
(TIF)

Figure S3 **^15^N-edited 1D NMR experiments demonstrate the solubility of amylin fibrils in DMSO.** (**A**) A 120 µM solution of ^15^N-amylin freshly dissolved in 95% DMSO/5% DCA. (**B**) Fibrils of ^15^N-amylin collected by sedimentation, lyophilized, and taken up in 95% DMSO/5% DCA. (**C**) Same as in B except pelleted fibrils were taken up in H_2_O. The lack of signal demonstrates the fibrils remain intact in H_2_O, in contrast to the spectrum in B where DMSO dissolves the fibrils. (**D**) Lyophilized supernatant from C taken up in H_2_O, showing amylin was incorporated into the fibrils, with negligible amounts of free monomers left in solution. Spectra were recorded at a temperature of 25°C and pH* 3.5. The spectra in C and D were collected with 8-times as many transients as B.
(TIF)
