# Supplementary figures and images for: Amide Proton Solvent Protection in Amylin Fibrils Probed by Quenched Hydrogen Exchange NMR
Source: PLoS One. 2013 Feb 15;8(2):e56467. doi: 10.1371/journal.pone.0056467 (PMC3574092; doi:10.1371/journal.pone.0056467)

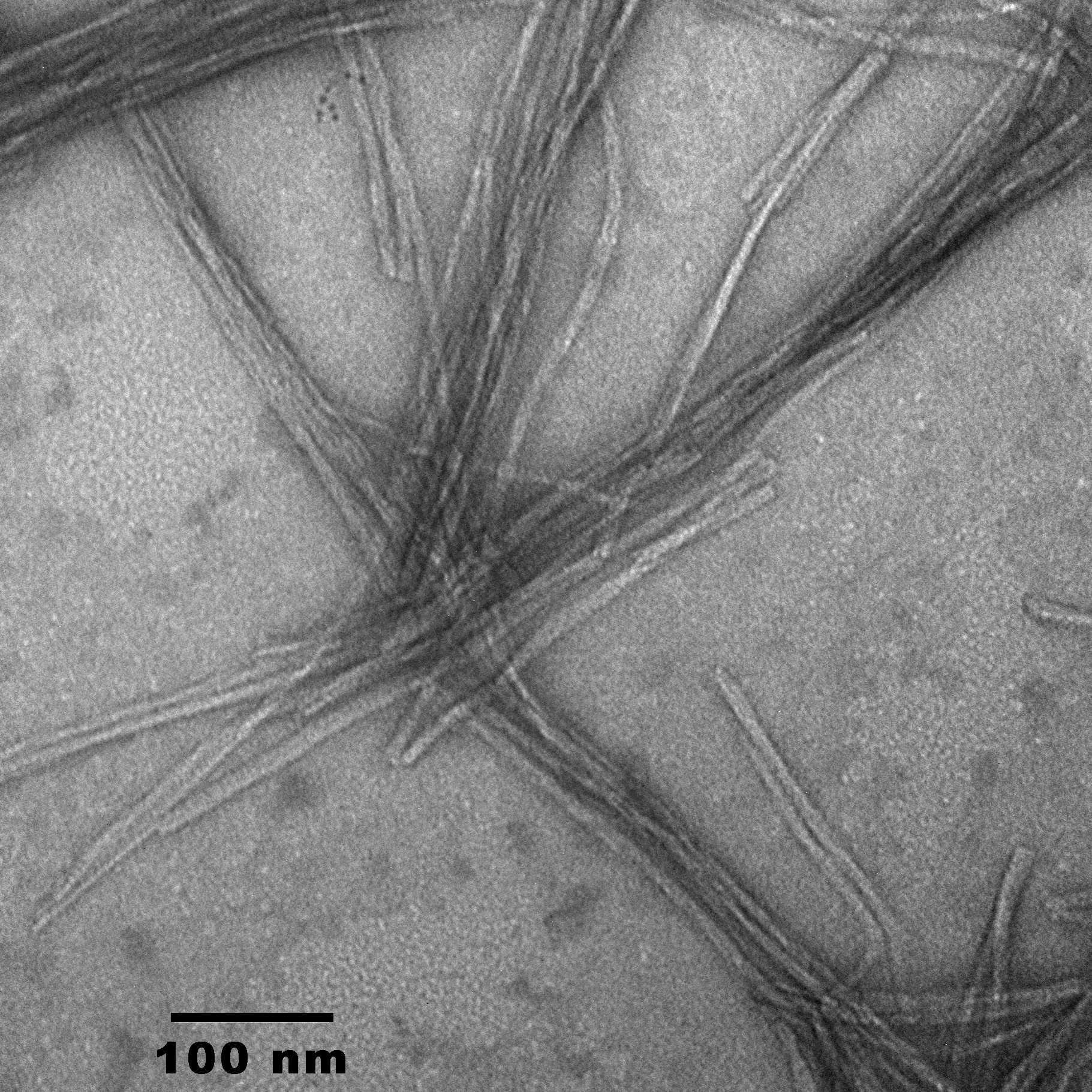

Supplement: Figure S2 — Electron micrograph of amylin fibrils. Fibrils of recombinant 15N-amylin were formed under the same conditions as the hydrogen exchange experiments. Fibrils were transferred to a 400-mesh carbon-coated grid, rinsed with H2O, and negatively stained with 1% uranyl acetate. Images were obtained on a FEI Tecnai G2 BioTWIN instrument that is part of the UConn electron microscopy facility. (TIF) [file pone.0056467.s002.tif]

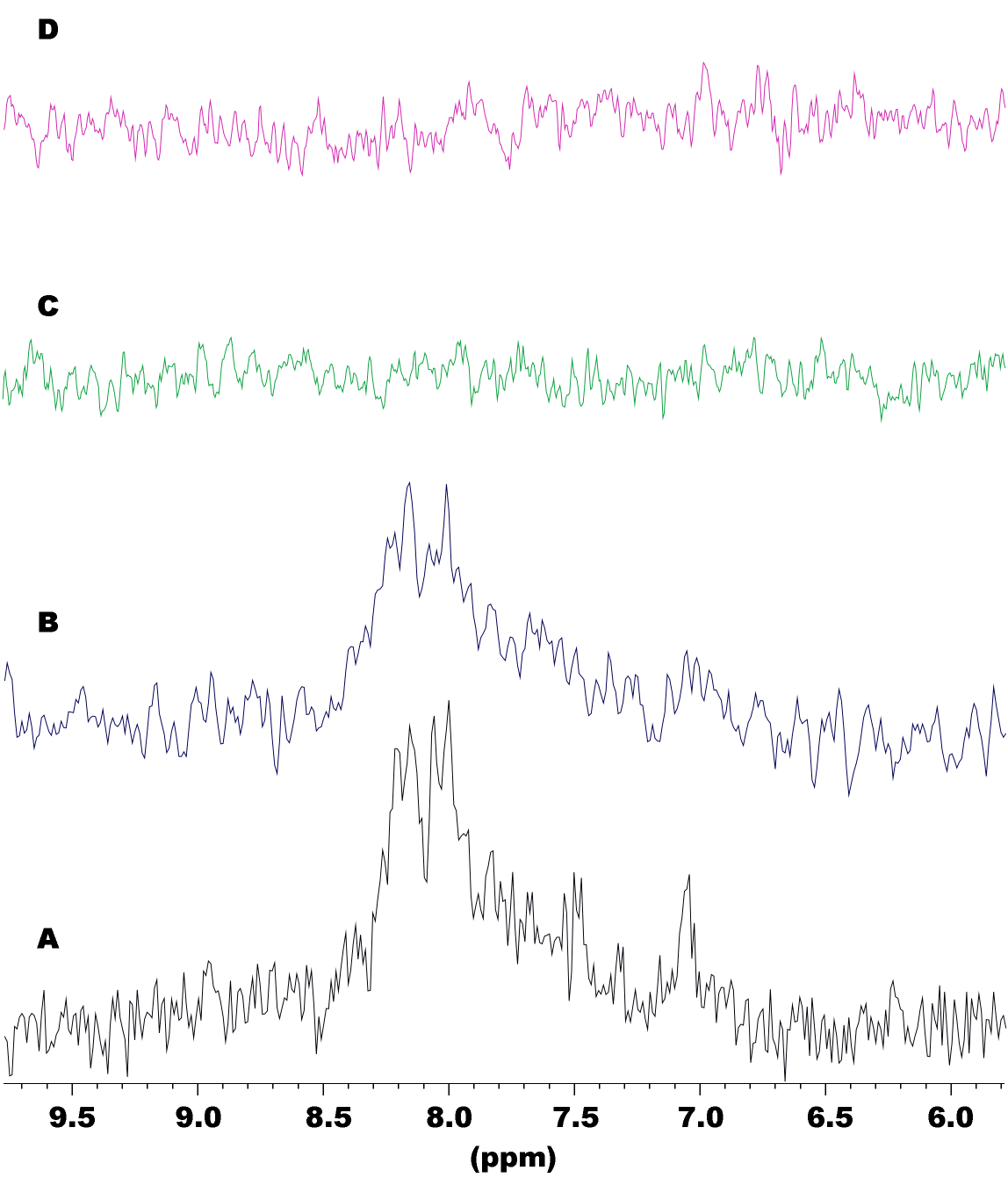

Supplement: Figure S3 — 15N-edited 1D NMR experiments demonstrate the solubility of amylin fibrils in DMSO. (A) A 120 µM solution of 15N-amylin freshly dissolved in 95% DMSO/5% DCA. (B) Fibrils of 15N-amylin collected by sedimentation, lyophilized, and taken up in 95% DMSO/5% DCA. (C) Same as in B except pelleted fibrils were taken up in H2O. The lack of signal demonstrates the fibrils remain intact in H2O, in contrast to the spectrum in B where DMSO dissolves the fibrils. (D) Lyophilized supernatant from C taken up in H2O, showing amylin was incorporated into the fibrils, with negligible amounts of free monomers left in solution. Spectra were recorded at a temperature of 25°C and pH* 3.5. The spectra in C and D were collected with 8-times as many transients as B. (TIF) [file pone.0056467.s003.tif]
